# Bendamustine-Based Regimens as Salvage Therapy in Refractory/Relapsed Multiple Myeloma Patients: A Retrospective Real-Life Analysis by the Polish Myeloma Group

**DOI:** 10.3390/jcm10235504

**Published:** 2021-11-24

**Authors:** Norbert Grzasko, Grzegorz Charlinski, Marta Morawska, Pawel Kicinski, Anna Waszczuk-Gajda, Joanna Drozd-Sokolowska, Edyta Subocz, Danuta Blonska, Malgorzata Razny, Agnieszka Druzd-Sitek, Jadwiga Holojda, Alina Swiderska, Lidia Usnarska-Zubkiewicz, Anna Masternak, Krzysztof Giannopoulos

**Affiliations:** 1 Department of Experimental Hematooncology, Medical University of Lublin, 20-400 Lublin, Poland; mmorawska79@gmail.com (M.M.); pawelkici@wp.pl (P.K.); krzysztof.giannopoulos@gmail.com (K.G.); 2 Department of Hematology, Medical Faculty, University of Warmia and Mazury in Olsztyn, 10-228 Olsztyn, Poland; grzegorzcharlinski0508@gmail.com; 3 Department of Hematology, Transplantation and Internal Medicine, Medical University of Warsaw, 02-091 Warsaw, Poland; annawaszczukgajda@gmail.com (A.W.-G.); johna.dr@poczta.fm (J.D.-S.); 4 Department of Internal Diseases and Hematology, Military Institute of Medicine, 04-141 Warsaw, Poland; suboczka@poczta.onet.pl; 5 Department of Hematology, Jan Biziel University Hospital No. 2, 85-168 Bydgoszcz, Poland; dankabl@poczta.onet.pl; 6 Department of Hematology and Internal Diseases, Ludwik Rydygier Specialistic Hospital, 31-826 Cracow, Poland; m.razny@wp.pl; 7 Department of Lymphoid Malignancies, Maria Sklodowska-Curie National Research Institute of Oncology, 00-001 Warsaw, Poland; adruzd@coi.waw.pl; 8 Department of Hematology, Specialistic City Hospital, 59-220 Legnica, Poland; J.Holojda@gmail.com; 9 Department of Hematology, University Hospital in Zielona Gora, 65-046 Zielona Gora, Poland; hematologia.zg@wp.pl; 10 Department of Hematology, Blood Neoplasms and Bone Marrow Transplantation, Wroclaw Medical University, 50-367 Wroclaw, Poland; lidiauz@wp.pl; 11 Department of Hematology, Specialist Hospital in Opole, 45-372 Opole, Poland; a_masternak@poczta.fm

**Keywords:** multiple myeloma, bendamustine, refractory/relapsed, salvage therapy, lenalidomide

## Abstract

Multiple myeloma (MM) is an incurable disease and patients become refractory to the treatment in the course of the disease. Bendamustine-based regimens containing steroids and other agents are among the therapeutic options offered to MM patients. Here, we investigated the safety and the efficacy of bendamustine used in patients with refractory/relapsed MM (RRMM). The patients were treated with bendamustine and steroids (*n* = 52) or bendamustine, steroids and immunomodulatory agents or proteasome inhibitors (*n* = 53). Response rates, progression-free survival (PFS), overall survival (OS) and frequency of adverse events were compared between both study groups. Most efficacy measurements were better in patients treated with three-drug regimens: overall response rate (55% versus 37%, *p* = 0.062), median PFS (9 months versus 4 months, *p* < 0.001), median OS survival (18 months versus 12 months, *p* = 0.679). The benefit from combining bendamustine and steroids with an additional agent was found in subgroups previously treated with both lenalidmide and bortezomib, with stem cell transplant and with more than two previous therapy lines. Toxicity was similar in both study groups and bendamustine-based therapies were generally well-tolerated. Our study suggests that bendamustine may be an effective treatment for patients with RRMM. Three-drug regimens containing bendamustine, steroids and novel agents produced better outcomes and had acceptable toxicity. The efficacy of bendamustine combined with steroids was limited.

## 1. Introduction

Multiple myeloma (MM) is a hematological neoplasm characterized by proliferation of monoclonal plasma cells in bone marrow, leading to secretion of monoclonal protein and subsequent complications: osteolytic lesions, hypercalcemia, anemia and renal function impairment [1]. The survival of patients with MM has significantly improved in recent decades after the introduction of new drugs, such as immunomodulatory agents (e.g., thalidomide, lenalidomide, pomalidomide), proteasome inhibitors (e.g., bortezomib, carfilzomib, ixazomib), histone deacetylase inhibitor-panobinostat, inhibitor of nuclear export-selinexor or monoclonal antibodies (e.g., daratumumab, isatuximab, elotuzumab) [2,3]. However, the disease remains uniformly incurable despite available therapies and patients who relapse after prior treatment or are refractory to treatment have a poor prognosis. Combination therapies including different cytotoxic agents and novel drugs are used in clinical practice in an attempt to treat patients with refractory/relapsed MM (RRMM).

One of the drugs used in the treatment of RRMM is bendamustine. Bendamustine is a bifunctional cytotoxic drug with a combined activity of alkylating agent and purine analog similar to cladribine and fludarabine. It exhibits cytotoxic activity against different human cancers, including ovarian and breast cancer and hematological malignancies such as lymphomas, chronic lymphocytic leukemia or MM. Importantly, in vitro studies showed that bendamustine had no cross-resistance with cyclophosphamide, melphalan, carmustine and cisplatin or cross-resistance was only partial. This finding makes bendamustine suitable for the treatment of patients with RRMM [4,5]. Bendamustine is approved in the United States by the Food and Drug Administration (FDA) for the treatment of indolent B-cell non-Hodgkin’s lymphoma and chronic lymphocytic leukemia. In Europe, the European Medicine Agency (EMA) approved bendamustine, additionally in combination with prednisone, for the treatment of patients with newly diagnosed MM who are not eligible for autologous stem cell transplant and have neuropathy, which contradicts the use of thalidomide or bortezomib. However, bendamustine is also widely used in patients with RRMM, both as monotherapy and in combination with one or two other myeloma-active agents. There are numerous studies reporting on the efficacy and safety of bendamustine in a patient setting: response rates usually ranged from 20 to 30% for monotherapy [6,7] to 35% for a combination of bendamustine with steroids [8] and more than 80% for triple-drug combinations, including bendamustine [9,10,11,12,13,14,15,16,17,18,19]. Drugs that were used in combination with bendamustine are steroids and thalidomide [9,10,11,12], lenalidomide [13,14,15,16] or bortezomib [17,18,19]. According to the National Comprehensive Cancer Network (NCCN) guidelines, bendamustine should be used in three-drug combinations with steroids and bortezomib or lenalidomide and only in certain circumstances, such as a monotherapy [20]. On the other hand, the European Society for Medical Oncology (ESMO) recommended bendamustine only in combination with prednisone in non-transplant eligible patients with clinical neuropathy at diagnosis following the official registration by EMA [21].

In the present study, we investigated the safety and efficacy of bendamustine used in two- and three-drug combinations in patients with RRMM treated in the centers of a Polish Myeloma Group. The aim of the study was to analyze the results of an unselected real-life cohort of patients treated routinely in daily practice.

## 2. Material and Methods

### 2.1. Patients Characteristics

In this multicenter retrospective study, a cohort of 105 patients with RRMM was treated with bendamustine-based regimens in 11 Polish sites in the years 2014–2019. One patient had plasma cell leukemia. Data on the presence of extramedullary plasmacytoma at relapse was not collected as it was not an exclusion criterion. The median age of patients was 64 and 41% were older than 65; 51% had ISS stage III, 30% had stage II and 19% stage I; median eGFR was 69 mL/min, 36% of patients had eGFR below 60 mL/min and 6% required dialysis; cytogenetic data were available in 20% of patients, 21% of them had del(17p), 19% had t(4;14) and 5% had t(14;16). Before being treated with bendamustine, patients received 1 to 8 treatment lines (median 3); 95% of patients were previously treated with immunomodulatory agents including thalidomide (89%) and lenalidomide (53%); 88% received proteasome inhibitors including bortezomib (79%) and carfilzomib (9%); 87% of patients were given therapies with both immunomodulators and proteasome inhibitors. Therapies with thalidomide included CTD (cyclophosphamide, thalidomide, dexamethasone), VTD (bortezomib, thalidomide, dexamethasone), MPT (melphalan, prednisone, thalidomide), TD (thalidomide, dexamethasone), TAD (thalidomide, doxorubicin, dexamethasone), T-VAD (thalidomide, vincristine, doxorubicin, dexamethasone), MT (melphalan, thalidomide) and monotherapy; therapies with lenalidomide included RD (lenalidomide, dexamethasone), RD with ixazomib and monotherapy; therapies with bortezomib included VTD, PAD (bortezomib, doxorubicin, dexamethasone), VMP (bortezomib, melphalan, prednisone), VCD (bortezomib, cyclophosphamide, dexamethasone), VD (bortezomib, dexamethasone) with panobinostat, VD, bortezomib with vorinostat and monotherapy; therapies with carfilzomib included KRD (carfilzomib, lenalidomide, dexamethasone) and KD (carfilzomib, dexamethasone). Detailed patient characteristics are summarized in Table 1.

### 2.2. Treatment Regimens

Patients were treated with different regimens containing bendamustine: 52 patients (49.5%) received bendamustine and steroids (group BendaSteroid) and 53 patients (50.5%) received three-drug regimens with bendamustine, steroid and immunomodulatory agent or proteasome inhibitor (group BendaSteroid + IMID/PI). The patients received the different treatments as a part of their usual care. Treatment cycles were given every 21 or 28 days, with bendamustine administration on days 1 and 2; bendamustine doses ranged between 60 mg/m^2^ and 150 mg/m^2^, with the most frequently given dose of 90 mg/m^2^ in 83 patients (79.0%). Steroids given to patients included oral prednisone, oral or intravenous dexamethasone, and intravenous methylprednisolone; the most frequent dose of steroid was 160 mg of dexamethasone or equivalent per cycle (range 30 to 320 mg). The third drug added to bendamustine and steroid was lenalidomide in 33 patients (31.4%), thalidomide in 12 patients (11.4%) and bortezomib in 8 patients (7.6%). All drugs were used at standard doses: lenalidomide 25 mg daily (or less in patients with renal function impairment) on days 1 to 21 in 28-day cycle; thalidomide 100 or 200 mg daily on days 1 to 21 or 28 depending on cycle duration; bortezomib 1.3 mg/m2 on days 1, 4, 8 and 11 in 21-day cycle or 1, 8, 15 and 22 in 28-day cycle.

### 2.3. Treatment Response and Toxicity Assessment

The efficacy of treatment with bendamustine-based regimens was assessed within the groups BendaSteroid and BendaSteroid + IMID/PI. Response criteria were based on the International Myeloma Working Group (IMWG) Uniform Response Criteria. Progression-free survival (PFS) was defined as the time from starting bendamustine therapy until disease progression or death from any cause; overall survival (OS) was defined as the time from bendamustine therapy first administration until death. Safety and toxicity of bendamustine therapy were assessed according to the National Cancer Institute Common Terminology Criteria for Adverse Events (CTCAE).

### 2.4. Statistical Analysis

The response to the treatment and toxicity were analyzed by descriptive statistics and differences between groups were compared by a Chi-square test. The analyses of PFS and OS were performed using Kaplan–Meier methodology; the differences in PFS and OS between study groups were assessed by the log-rank test; *p*-values < 0.05 were considered statistically significant. Calculation of follow-up time was performed using a reverse Kaplan–Meier method. All statistical calculations were carried out with the use of packages Statistica (StaSoft Inc, Tulusa, OK, USA) and GraphPad Prism (GraphPad Software Inc, San Diego, CA, USA).

## 3. Results

### 3.1. Response to Treatment

The median number of cycles given to patients was 4 (range 1–8) and there was no difference between patients treated with BendaSteroid and BendaSteroid + IMID/PI. The overall response rate (ORR) to bendamustine-based therapies in all patients was 46% including 8% of complete responses (CR), 9% of very good partial responses (VGPR), 30% of partial responses (PR), 35% of stable disease (SD) and 19% of progressive disease (PD). The median time to first response (at least PR) was 2 months (range 1–6 months) and to best response was 3 months (range 1–13 months). ORR was higher in subjects treated with BendaSteroid + IMID/PI than in those treated with BendaSteroid, although the difference was not statistically significant (55% versus 37%, *p* = 0.062). Median time to first and best response was similar in both study groups: to first response 2 months in treated with BendaSteroid (range 2–6) and 2 months in treated with BendaSteroid + IMID/PI (range 1–4); to best response 2 months (range 2–6) and 3 months (range 2–13), respectively.

The rate of good quality responses was significantly higher in patients treated with BendaSteroid + IMID/PI: CR rate was 13% versus 2% (*p* = 0.029) and VGPR or better rate was 25% versus 8% (*p* = 0.019). On the other hand, the rate of SD was higher in patients treated with BendaSteroid (46% versus 25%, *p* = 0.20). Detailed response rates are presented in Table 2.

### 3.2. Progression-Free Survival

**Figure 1:**
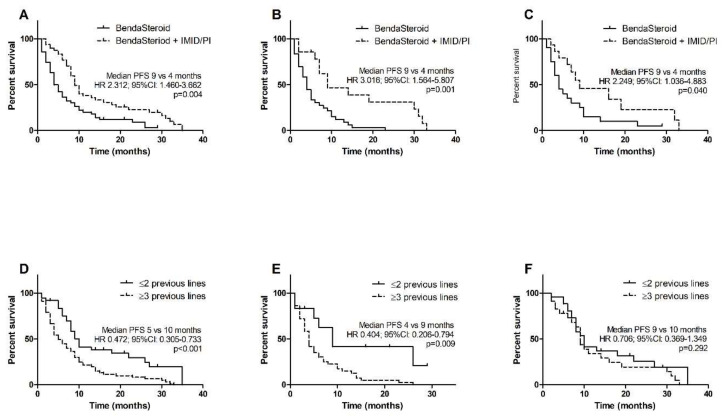
Kaplan–Meier estimates of progression-free survival (PFS): (**A**) all patients; (**B**) subgroup of patients previously treated with lenalidomide and bortezomib; (**C**) subgroup of patients after ASCT; (**D**) impact of previous therapies number in all patients; (**E**) impact of previous therapies number in patients treated with bendamustine and steroid; (**F**) impact of previous therapies number in patients treated with bendamustine, steroid and IMID/PI.

The median follow-up in the whole group was 35 months. The median PFS in the whole study group was 9 months. Patients previously treated with three or more lines of therapy had poorer PSF than those who received one or two lines (5 months versus 10 months, HR 0.472, 95% CI 0.305–0.733, *p* < 0.001). The median PFS was significantly longer in subjects treated with BendaSteroid + IMID/PI than in those given BendaSteroid (9 months versus 4 months, HR 2.312, 95% CI 1.460–3.662, *p* = 0.004). The difference between patients after ≥3 therapy lines and after 1–2 therapy lines was found to be significant in the BendaSteroid group (4 months versus 9 months, HR 0.404, 95% CI 0.206–0.794, *p* = 0.009), but not in the triplet group (9 months versus 10 months, HR 0.706, 95% CI 0.369–1.349, *p* = 0.292). Benefit from adding a third drug to bendamustine-steroid backbone was found regardless of the type of previous treatment; in patients after both bortezomib and lenalidomide the median PFS was 9 months in the triple-drug group and 4 months in BendaSteroid group (HR 3.016, 95% CI 1.564–5.807, *p* = 0.001); in patients after previous autologous stem cell transplant (ASCT) it was also 9 months and 4 months, respectively (HR 2.249, 95% CI 1.036–4.883, *p* = 0.040). Kaplan–Meier estimates of PFS are shown in Figure 1.

### 3.3. Overall Survival

**Figure 2:**
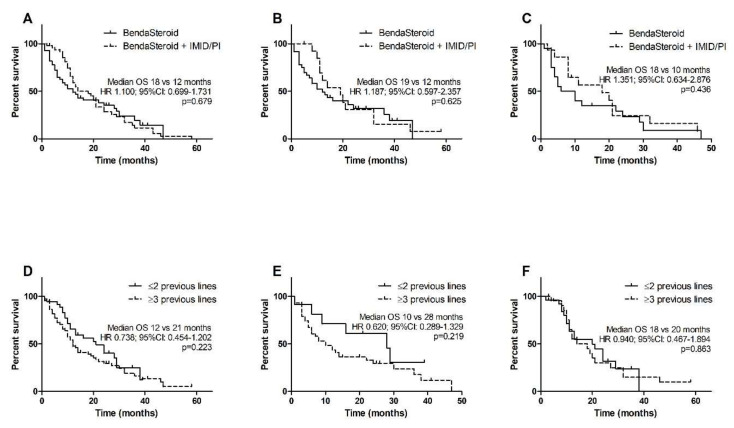
Kaplan–Meier estimates of overall survival (OS): (**A**) all patients; (**B**) subgroup of patients previously treated with lenalidomide and bortezomib; (**C**) subgroup of patients after ASCT; (**D**) impact of previous therapies number in all patients; (**E**) impact of previous therapies number in patients treated with bendamustine and steroid; (**F**) impact of previous therapies number in patients treated with bendamustine, steroid and IMID/PI.

The median OS in all patients was 14 months. Similarly, to PFS, patients previously treated with ≥3 lines of therapy had poorer OS than treated with 1–2 lines, however the difference was not statistically significant (12 months versus 21 months, HR 0.738, 95% CI 0.454–1.202, *p* = 0.223). The median OS was longer in subjects treated with BendaSteroid + IMID/PI than in those given BendaSteroid, although the difference was not statistically significant (18 months versus 12 months, HR 1.100, 95% CI 0.699–1.731, *p* = 0.679). The difference between patients after ≥3 therapy lines and after 1–2 therapy lines was notable but not significant in BendaSteroid group (10 months versus 28 months, HR 0.620, 95% CI 0.289–1.329, *p* = 0.219) and inconsiderable in the triplet group (18 months versus 20 months, HR 0.940, 95% CI 0.467–1.894, *p* = 0.863). The addition of a third drug to bendamustine and steroid trended towards prolonged OS in patients who were previously treated with both bortezomib and lenalidomide and who underwent ASCT, although for both cohorts the differences were not statistically significant. In a cohort after previous bortezomib and lenalidomide the median OS was 19 months in the triple-drug group and 12 months in BendaSteroid group (HR 1.187, 95% CI 0.597–2.357, *p* = 0.625); in patients after previous ASCT it was 18 months and 10 months, respectively (HR 1.351, 95% CI 0.634–2.876, *p* = 0.436). Kaplan–Meier estimates of OS are shown in Figure 2.

### 3.4. Patients with High-Risk Cytogenetics

Results of cytogenetic studies were available in 21 (20%) patients; 8 of them had abnormalities that confer poor prognosis such as del(17p13), t(4;14) and t(14;16). Patients with high-risk cytogenetics had shorter median PFS (4 months versus 9 months, HR 0.366, 95% CI 0.114–1.174, *p* = 0.091) and OS (6 months versus 21 months, HR 0.157, 95% CI 0.042–0.592, *p* = 0.006) than those with standard-risk results. Analysis of survival in both cytogenetic subgroups showed an advantage of BendaSteroid + IMID/PI. In the high-risk subgroup median PFS and OS were longer when patients were given bendamustine-based triplets (4.5 versus 4 months and 11 versus 5 months, respectively). Similar results were found in the standard-risk group (median PFS 13 versus 5 months and median OS 21 versus 16 months). Due to the small number of subjects, all differences were not statistically significant, except PFS in the standard-risk patients (HR 5.627, 95% CI 1.555–20.360, *p* = 0.009).

### 3.5. Toxicity and Tolerability of Bendamustine-Based Therapies

The treatment with bendamustine-based therapies was generally well-tolerated and the frequency of adverse events was similar in both study groups. Hematological toxicity occurred in 68% of patients in BendaSteroid group and in 71% of patients in the triple-drug group. The most common was neutropenia which was found in BendaSteroid group at any grade in 54% and at grade 3 or more in 29% of patients; in BendaSteroid + IMID/PI group it occurred in 60% and 47% of patients, respectively; both differences were not statistically significant. The occurrence of anemia and thrombocytopenia was similar within study groups, both any grade and grade 3 or more. Non-hematological toxicity was found in 70% of patients treated with BendaSteroid and in 55% of patients treated with bendamustine-based triplets. The most common were infections which were diagnosed more commonly in BendaSteroid group (52% versus 32%), although the difference was not statistically significant. Other toxicities were found in few patients. The occurrence of adverse events is summarized in Table 3.

## 4. Discussion

Multiple myeloma remains an incurable disease despite advances in the treatment and new drugs available. It is particularly difficult to obtain a response in patients with RRMM after a few lines of therapy. Our study demonstrated that bendamustine, especially in three-drug combinations, may be effective and safe in this group of patients.

Studies investigating bendamustine in patients with RRMM published so far showed various results, mostly depending on different drugs which were combined with bendamustine. Single agent bendamustine was not very effective drug when used in heavily pretreated MM patients. In one of the published studies the response rate was 20% (partial response or better) with no complete responses, further 39% of patients had a minimal response, the median event-free survival (EFS) was 7 months, and the median OS was 17 months. Interestingly, patients who received a concomitant steroid had shorter OS (13 months) [6]. In another study with RRMM patients who previously received alkylators, steroids, IMiDs and bortezomib, the ORR (PR or better) was 30%, including 2% CR, the median PFS was 9.3 months, and the median OS was 12.4 months [7]. One more report supported the above findings showing the ORR of 36% with 3% of VGPR, the median PFS of 7 months and the median OS of 17 months. The study included patients treated with bendamustine both in monotherapy and in combination with steroids [22]. There was only one study that demonstrated better results obtained with bendamustine as monotherapy: in a group of 31 patients relapsed after autologous stem cell transplant the ORR was 55% and the median PFS 26 weeks in all patients and 36 weeks in those receiving higher doses of bendamustine (90–100 mg/m^2^) [23]. The addition of steroids seems not to improve the efficacy of bendamustine significantly. In one study 65 heavily pretreated patients (5 or more therapy lines in 63%) were given bendamustine with prednisone and results were only slightly better than in studies with bendamustine monotherapy: the ORR was 35%, the median PFS was 3.1 months and OS 5.5 months [8].

Our results are in line with the above-mentioned studies. We obtained the ORR of 37%, the median PFS of 4 months and the median OS of 12 months in patients treated with BendaSteroid. The results were strongly influenced by the number of previous therapy lines and were significantly better in patients who received no more than two lines before bendamustine. This finding suggests that BendaSteroid should not be recommended for really heavily pre-treated patients with RRMM.

Numerous studies with triple-drug regimens containing bendamustine used in patients with RRMM showed better efficacy than bendamustine, such as monotherapy or with steroids only. However, the results of these studies were divergent and showed the ORR from 26% to 86%, the median PFS from 3 months to 48% surviving without progression after 18 months and the median OS from 7.2 months to 50 months. In one study investigating the combination of bendamustine, thalidomide and prednisone in 28 patients with RRMM, the response rate was 86%, the median PFS 11 months and the median OS 19 months [9]. Another one showed much worse results: clinical benefit defined as SD or better was 61%, but ORR was only 26%, the median PFS was 3 months and the median OS 13 months. However, the median number of previous therapy lines given to patients was 5 and almost all enrolled subjects had previously received bortezomib, lenalidomide and ASCT [10]. One more study involving heavily pre-treated patients who were previously given lenalidomide and bortezomib reported similar results with the median PFS of 4 months and OS of 7.2 months [11]. All above-mentioned studies investigated small groups of patients (up to 30), which possibly influenced the results. The biggest study with BTD regimen (bendamustine, thalidomide, dexamethasone) was conducted in the group of 94 patients and showed a response rate of 46.3% and a median PFS of 7.5 months [12].

A similar discrepancy regarding the treatment results can be found in studies investigating the combination of bendamustine, dexamethasone and lenalidomide. There are studies reporting a response rate of more than 70% or even 80% [13,14]. One of these studies involved patients after only one therapy line and the response rate was 88.9% with 51% of patients achieving VGPR or better, the median PFS was 18.6 months, and 2-year OS was 76% [13]. In studies with more pre-treated patients given bendamustine, dexamethasone and lenalidomide the response rate was about 50% and the median PFS no more than 10 months [15,16].

Patients treated with bendamustine and dexamethasone with bortezomib had better results when compared to combinations with thalidomide or lenalidomide [17,18,19]. However, the studies reporting such results involved less pre-treated patients, although the groups of patients were quite big exceeding 70 subjects. In two studies the patients previously received two median therapy lines. The response rates were 60.8% and 69%, the median PSF 9.7 months and 11 months, the median OS 25.6 and 50 months [17,18]. In the study with patients who had never received bortezomib before, the ORR was 71.5%, the median PFS was 16.5 months and 1-year OS 78% [19].

Our study showed similar results regarding ORR, PFS and OS to the above-mentioned reports, both in the group treated with bendamustine and steroid, and in the group treated with bendamustine-based triplet therapies. The response rate was higher in the triplet group (55% versus 37%), including significantly higher rates of CR (13% versus 2%) and at least VGPR (25% versus 8%). Our data also indicated that bendamustine-based triplet therapies were significantly more efficient than a combination of bendamustine and steroid in terms of survival. The median PFS was longer in the triplet group (9 versus 4 months) and a similar difference was found in subgroups of patients previously treated with bortezomib/lenalidomide and given ASCT. Patients after the previous 1–2 lines of therapy had significantly longer PFS than more pre-treated subjects when the whole group and the BendaSteroid group were analyzed, but such a difference was not observed in the triplet group. In our study the patients treated with bendamustine and steroid more frequently received previous lenalidomide and bortezomib than the patients treated with triplets (71% versus 26%). However, the previous therapy with these drugs was not found to have an adverse effect on outcome in the triplet group in contrast to the BendaSteroid group, which indicated that better outcomes in the triplet group were not associated with the difference in previous treatment. The difference in OS between our study groups was similar to observed in PFS. The treatment with triple therapies was associated with the longer OS when we analyzed all patients, patients after previous lenalidomide and bortezomib and patients after ASCT. Similar to PFS, an adverse effect of 3 or more previous therapy lines was found only in patients treated with bendamustine and steroid, but not with triplets. All differences in OS were not statistically significant, probably due to the effect of various subsequent therapies.

Recently, some reports on bendamustine-based combinations were published including newer immunomodulatory agents or proteasome inhibitors. The results of an open-label phase I/II trial of pomalidomide, bendamustine and dexamethasone showed that this combination was an effective treatment option in patients with RRMM. The ORR was 61% and the median PFS and OS were 9.6 months and 21.3 months, respectively [24]. In another phase I/II trial, RRMM patients treated with ixazomib, bendamustine and dexamethasone had the median PFS of 5.2 months, the median OS of 23.2 months and the ORR was 61% [25]. The most promising results were found in a study with carfilzomib, bendamustine, and dexamethasone, although the number of participating RRMM patients was only 17. The ORR reported by authors was 88%, the median PFS 15.1 months and the median OS 56.3 months [26]. Interestingly, the same combination used in newly diagnosed MM produced the OR of 100%, the median PFS of 56 months, and the median OS was not reached [27].

Regarding safety profile, in most reports, bendamustine-based therapies were well-tolerated, and most adverse events were hematological [6,7,8,9,10,11,12,13,14,15,16,17,18,19]. Non-hematological adverse events were either associated with hematological complications, such as infections due to neutropenia, or related to other drugs, such as hyperglycemia due to steroids [16] or neuropathy due to bortezomib [19]. In our study, there were no significant differences in the frequency of adverse events between patients treated with bendamustine and steroids and subjects given these drugs in combination with one more agent. Both regimens were similarly well-tolerated and the only pronounced difference regarding neutropenia was not statistically significant.

## 5. Conclusions

Our data confirmed that bendamustine-based three-drug regimens were effective and well-tolerated in the treatment of patients with RRMM, whereas the combination of bendamustine and steroids had limited activity. Particularly, patients previously treated with more than two therapy lines or given lenalidomide and bortezomib did not benefit from a two-drug bendamustine combination. The results of our study suggested that bendamustine should be used in three-drug regimens.

To our knowledge, this is the first real-life study showing an advantage of bendamustine-based triple-drug over two-drug regimens in patients with RRMM.

## Figures and Tables

**Table 1:** Patient characteristics.

	All Patients	Bendamustine + Steroid	Bendamustine + Steroid + Thal/Len/Bort
	*n* = 105	*n* = 52	*n* = 53
Sex			
male	50 (48%)	24 (46%)	26 (49%)
female	55 (52%)	28 (54%)	27 (51%)
Age			
median (range)	64 (45–84)	64 (45–82)	65 (49–84)
>65 years	43 (41%)	22 (42%)	21 (40%)
Monoclonal protein type			
IgG	68 (65%)	35 (67%)	33 (62%)
IgA	25 (24%)	12 (23%)	13 (25%)
LCD	12 (11%)	5 (10%)	7 (13%)
Light chain			
kappa	61 (58%)	31 (60%)	30 (57%)
lambda	44 (42%)	21 (40%)	23 (43%)
ISS stage			
I	20 (19%)	11 (21%)	9 (17%)
II	31 (30%)	16 (31%)	15 (28%)
III	54 (51%)	25 (48%)	29 (55%)
Cytogenetics			
unknown	84 (80%)	43 (83%)	41 (77%)
del(17p13)	5 (24%) ^1^	3 (33%) ^1^	2 (17%) ^1^
del(13q14)	3 (14%) ^1^	1 (11%) ^1^	2 (17%) ^1^
t(4;14)(p16;q32)	4 (19%) ^1^	1 (11%) ^1^	3 (25%) ^1^
t(14;16)(q32;q23)	1 (5%) ^1^	1 (11%) ^1^	0 (0%) ^1^
Serum monoclonal protein	23.9 (0.87–95.6)	22.7 (0.87–95.6)	25.2 (1.37–84.56)
(g/L; median, range)			
Bone marrow plasma cells	46.5 (7–90)	48.0 (10–90)	42.5 (7–85)
(%; median, range)			
Albumin	3.6 (2.01–5.33)	3.6 (2.01–5.33)	3.6 (2.01–5.33)
(g/dL; median, range)			
Beta-2-microglobulin	4.93 (1.1–40)	4.65 (2.5–36)	5.11 (1.1–40)
(mg/L; median, range)			
LDH	211 (75–2205)	238 (75–1872)	281 (107–2205)
(IU/L; median, range)			
Creatinine	0.965 (0.4–9.33)	1.053 (0.64–7.83)	0.924 (0.4–9.33)
(mg/dL; median, range)			
eGFR	69 (5.37–185.9)	68 (5.37–152.7)	71 (9.5–185.9)
(mL/min; median, range)			
Calcium	2.37 (1.79–4.94)	2.45 (2.08–4.94)	2.29 (1.79–4.63)
(mmol/L; median, range)			
Neutrophils	3.08 (0.59–8.87)	3.42 (1.15–8.87)	2.84 (0.59–7.59)
(G/L; median, range)			
Hemoglobin	10.25 (6.6–14.7)	10.13 (6.6–14.7)	10.37 (6.9–13.6)
(g/dL; median, range)			
Platelets	159 (22–409)	149 (22–378)	161 (29–409)
(G/L; median, range)			
Previous treatment			
Number of lines (median, range)	3 (1–8)	3 (1–8)	3 (1–6)
IMID	100 (95%)	51(98%)	49 (92%)
Thalidomide	93 (89%)	48 (92%)	45 (85%)
Lenalidomide	56 (53%)	38 (73%) ^2^	18 (34%) ^2^
PI	92 (88%)	48 (92%)	44 (83%)
Bortezomib	83 (79%)	45 (87%)	38 (72%)
Carfilzomib	9 (9%)	3 (6%)	5 (9%)
Lenalidomide + Bortezomib	51 (49%)	37 (71%) ^2^	14 (26%) ^2^
IMID + PI	91 (87%)	47 (90%)	44 (83%)
ASCT	33 (31%)	16 (31%)	17 (32%)

^1^ percentage of patients with available cytogenetic test ^2^ *p* < 0.05 LCD—light chain disease; ISS—International Staging System; LDH—*lactate dehydrogenase*; eGFR—estimated glomerular filtration rate; IMID—immunomodulatory drug; PI—proteasome inhibitor; ASCT—autologous stem cell transplant.

**Table 2:** Response to bendamustine-based regimens.

	All Patients *n* = 105	Bendamustine + Steroid *n* = 52	Bendamustine + Steroid + Thal/Len/Bort *n* = 53
ORR	48 (46%)	19 (37%)	29 (55%)
CR	8 (8%)	1 (2%) *	7 (13%) *
VGPR	9 (9%)	3 (6%)	6 (11%)
CR + VGPR	17 (16%)	4 (8%) *	13 (25%) *
PR	31 (30%)	15 (29%)	16 (30%)
SD	37 (35%)	24 (46%)	13 (25%)
PD	20 (19%)	9 (17%)	11 (21%)

* *p* < 0.05 ORR—overall response rate; CR—complete response; VGPR—very good partial response; PR—partial response; SD—stable disease; PD—progressive disease.

**Table 3:** Adverse events during bendamustine-based therapies. All differences in toxicity occurrence between study groups were not statistically significant (*p* > 0.05).

	Bendamustine + Steroid *n* = 52		Bendamustine + Steroid + Len/Thal/Bort *n* = 53	
	All grades	Grade ≥3	All grades	Grade ≥3
Neutropenia	28 (54%)	15 (29%)	32 (60%)	25 (47%)
Anemia	18 (35%)	11 (21%)	21 (40%)	6 (11%)
Thrombocytopenia	17 (33%)	10 (19%)	15 (28%)	10 (19%)
Infection	27 (52%)	10 (19%)	20 (38%)	5 (11%)
Gastrointestinal toxicity	5 (10%)	0 (0%)	3 (7%)	2 (4%)
Thromboembolism	1 (2%)	1 (2%)	2 (4%)	1 (2%)
Neuropathy	0 (0%)	0 (0%)	1 (2%)	0 (0%)
Renal function impairment	2 (4%)	0 (0%)	1 (2%)	0 (0%)
Hepatic toxisity	0 (0%)	0 (0%)	1 (2%)	0 (0%)
Hypertension	1 (2%)	1 (2%)	0 (0%)	0 (0%)

## Data Availability

Data are contained within the article. Any additional data are available from the corresponding author upon reasonable request.
